# Distributions of Platelet-Derived Growth Factor Receptor-α Positive Cells and Interstitial Cells of Cajal in the Colon of Rats with Diabetes Mellitus Type 2

**DOI:** 10.3390/medicina59020308

**Published:** 2023-02-07

**Authors:** Aleksandra Ivana Veličkov, Branka Djordjević, Milica Lazarević, Asen Veselin Veličkov, Vladimir Petrović, Marko Jović, Tijana Denčić, Goran Radenković

**Affiliations:** 1 Department of Histology and Embryology, Faculty of Medicine, University of Niš, 18000 Niš, Serbia; 2 Department of Biochemistry, Faculty of Medicine, University of Niš, 18000 Niš, Serbia; 3 Clinic for Orthopedic Surgery and Traumatology, University Clinical Centre Niš, 18000 Niš, Serbia; 4 Department of Pathology, Faculty of Medicine, Clinical Centre Niš, University of Niš, 18000 Niš, Serbia

**Keywords:** diabetes mellitus type 2, diabetic gastroenteropathy, interstitial cells, colon

## Abstract

*Background and Objectives*: Diabetic gastroenteropathy (DG) is a common complication of diabetes mellitus type 2. Interstitial cells are non-neural cells of mesenchymal origin inserted between nerve elements and smooth muscle cells, necessary for normal function and peristaltic contractions in the gastrointestinal (GI) tract. There are at least two types of interstitial cells within the GI muscle layer—interstitial cells of Cajal (ICC) and interstitial platelet-derived growth factor receptor α-positive cells (IPC). The mechanism of diabetic gastroenteropathy is unclear, and interstitial cells disorders caused by metabolic changes in diabetes mellitus (DM) could explain the symptoms of DG (slow intestinal transit, constipation, fecal incontinence). The aim of this study was to identify PDGFRα and c-kit immunoreactive cells in the colon of rats with streptozotocin–nicotinamide-induced diabetes mellitus type 2, as well as to determine their distribution in relation to smooth muscle cells and enteric nerve structures. *Materials and Methods*: Male Wistar rats were used, and diabetes type 2 was induced by an intraperitoneal injection of streptozotocin, immediately after intraperitoneal application of nicotinamide. The colon specimens were exposed to PDGFRα and anti-c-kit antibodies to investigate interstitial cells; enteric neurons and smooth muscle cells were immunohistochemically labeled with NF-M and desmin antibodies. *Results*: Significant loss of the intramuscular ICC, myenteric ICC, and loss of their connection in intramuscular linear arrays and around the ganglion of the myenteric plexus were observed with no changes in nerve fiber distribution in the colon of rats with diabetes mellitus type 2. IPC were rarely present within the colon muscle layer with densely distributed PDGFRα+ cells in the colon mucosa and submucosa of both experimental groups. In summary, a decrease in intramuscular ICC, discontinuities and breakdown of contacts between myenteric ICC without changes in IPC and nerve fibers distribution were observed in the colon of streptozotocin/nicotinamide-induced diabetes type 2 rats.

## 1. Introduction

A wide spectrum of gastrointestinal motility disorders occur in up to 50–70% of patients with diabetes mellitus type 2 [1]. Diabetic gastroenteropathy is a common complication of diabetes mellitus that can manifest as gastroparesis, slowed intestinal transit, constipation, diarrhea, fecal incontinence, dysphagia, heartburn, abdominal discomfort or pain, nausea and vomiting [2]. The mechanism of diabetic gastroenteropathy development is complex and multifactorial, and understanding it is essential for the evolving therapeutical procedures that can be used to solve this common problem. Diabetes can affect all the cells involved in the regulation of peristalsis, such as enteric neurons, smooth muscle cells, vascular endothelium within myenteric plexus ganglia, and specific interstitial cells. Although interstitial cells represent only 5% of the muscle layer cell populations in the gastrointestinal tract (GIT), they are necessary for the establishment and adequate functioning of gastrointestinal motility [3]. Initiation and regulation of peristalsis is a complex process that involves several interstitial cell types in addition to enteric nerves and smooth muscle cells. There are at least two types of interstitial cells within the GIT muscle layer—interstitial cells of Cajal (ICC) and interstitial platelet-derived growth factor receptor α-positive cells (IPC), also called “fibroblast-like” or “ICC-like cells” [4,5]. This heterogenous interstitial cell population of mesenchymal origin is inserted between nerve elements and smooth muscle cells where they form connections with each other and with surrounding smooth muscle cells with a significant role in conducting electrical signals and regulating muscle excitability [3,6]. The collaboration and networking of the mentioned structures represents the histophysiological basis for peristaltic contractions and normal functioning of the gastrointestinal tract.

Platelet-derived growth factors (PDGFs) are mitogenic for many cells of mesenchymal origin, such as fibroblasts and smooth muscle cells [7]. PDGF signalization is important in organogenesis [8], while in adult cells, platelet-derived growth factor receptor alpha (PDGFRα) expression plays a role in the pathophysiology of various disorders, including gastrointestinal motility disorder [9]. Immunohistochemical labeling of PDGFRα enables identification of IPC, PDGFRα-positive interstitial “fibroblast-like” cells [10]. Although these cells share the same location with ICC throughout the GIT of mice, primates and humans, they are different and supposed to have modulatory and inhibitory regulation of intestinal contraction [10,11,12,13,14].

The primary ligand for a transmembrane protein c-kit is the stem cell factor (SCF). This SCF-KIT signaling is essential for the survival, proliferation and differentiation of KIT-expressing cells, such as mast cells, melanocytes and ICC [15,16]. In addition to the identification of ICC by c-kit immunohistochemistry, electron microscopy analysis has revealed distinct morphological features based on location within the muscular GIT layer [17,18,19]. ICC are organized in the form of two-dimensional or three-dimensional networks and bundles of interconnected cells, which are classified based on their morphology, location, and function into several subtypes: intramuscular, myenteric, submucosal, subserous, and septal ICC cells. The primary role of ICC cells located around the myenteric plexus ganglion (ICC-MP) is to generate slow waves of depolarization which determine the progression and duration of the contraction of the gastrointestinal tract musculature. These cells are termed pacemakers of the gastrointestinal musculature [20,21]. The intramuscular ICC subtype (ICC-IM) serves as a mediator of cholinergic and nitrergic neurotransmission [22,23], and also participates in the afferent signaling and integration of sensory–motor function as an element of the afferent branch of the gastrointestinal reflex [24]. ICC have a role in sensory transduction of mechanical stimuli, in the feedback that affect motility, and serve as stretch receptors [25]. Loss and dysfunction of ICC have been demonstrated in numerous motility disorders [26,27,28].

The aim of this study was to identify PDGFRα and c-kit immunoreactive cells in the colon of rats with streptozotocin/nicotinamide-induced diabetes mellitus type 2, as well as to determine their distribution in relation to smooth muscle cells and enteric nerve structures.

## 2. Materials and Methods

### 2.1. Animals

Ten-weeks-old colon sections from male Wistar rats weighing 230–250 g were used in the present study. The study was carried out in accordance with the National Guide for the Care and Use of Laboratory Animals, and all experimental protocols have been previously approved by the Ethics Committee of the Faculty of Medicine, University of Nis, Serbia (permit number 12–519/7). The study was performed within the Internal project no.38/20 of the Faculty of Medicine in Nis at the Research Center for Bio-medicine and Department for Histology and Embryology. During the experiments, the rats were housed in plastic cages in a controlled environment with unlimited access to food and water. The animals were randomly divided into two groups: diabetic and age-matched controls. Diabetes mellitus type 2 was induced by an intraperitoneal injection of freshly prepared streptozotocin (Sigma Aldrich, SAD) at a dose of 45 mg/kg in ice-cold 0.1 mol/L citrate buffer (pH 4.5) following an intraperitoneal injection of nicotinamide (Sigma Aldrich, SAD) at a dose of 110 mg/kg in saline solution after an overnight fasting according to the modified model already described by Masiello et al. [29]. Hyperglycemia was verified on the 3rd and 7th day from the tail vein after streptozotocin/nicotinamide (STZ–NA) administration using glucose meter Accu-check Performa (Roche Diagnostics, USA). The animals with glucose level >8.3 mmol/L were considered diabetic, while the rest of the animals were excluded from the experiment so that 10 animals entered the experiment per group (*n* = 10). Serum insulin levels were measured with a commercial rat enzyme-linked immunosorbent assay (ELISA) kit using rat insulin as the standard (Mercodia, Upsala, Sweden; catalog number 10-1250-01). Six weeks after the onset of diabetes type 2, the animals were sacrificed by exsanguination after bilateral thoracotomy in deep anesthesia (ketamine hydro-chloride, 100 mg/kg body weight).

Colon samples (caecum, proximal, and distal colon) were removed via abdominal incision immediately after exsanguination, emptied with saline solution, fixed in buffered formalin (10%) for 24 h and paraffin-embedded by routine procedure.

### 2.2. Hematoxylin and Eosin (he) Staining Method

The tissue slides were rehydrated in descending series of ethanol (100%, 96%, 76% of ethanol), and distilled water. Hematoxylin was applied for 8 min and the stain was differentiated into the tap water, followed by washing of the tissue slides in distilled water. In the next step, eosin was applied for 20 min, followed by rinsing in the distilled water. The tissue slides were dehydrated in the ascending series of alcohols (76%, 96% and 100% of ethanol), cleared in xylene and mounted using Canada Balsam.

### 2.3. Immunohistochemistry

The 4–5 µm thick sections were exposed to PDGFRα (Abcam, Cambridge, United Kingdom) and anti-c-kit (CD 117) antibodies (Abcam, Cambridge, UK) to investigate IPC and ICC; enteric neurons and smooth muscle cells were immunohistochemically labeled with NF m antibodies (Abcam, Cambridge, United Kingdom) and anti-desmin antibodies (Dako, Denmark), respectively. After deparaffinization in a thermostat at 60 °C in xylol and rehydration in a descending series of ethanol rinses and distilled water (100% ethanol—1 min, 96% ethanol—1 min, distilled water—5 min), the sections were pre-treated for immunohistochemical analysis with 30 min heat-induced epitope retrieval in a commercial solution EnVisiontmFLEXtarget retrieval DM 828, (50×), (Dako, Denmark). Endogenous peroxidase was blocked by a 3% H_2_O_2_ solution for 10 min at room temperature. The primary antibodies were diluted in commercial antibody diluent AntybodyDiulentEnVisiontmFLEX DM 830 (Dako, Denmark). The primary antibodies used in the research, their respective dilutions and incubation are listed in Table 1. After secondary antibody administration (EnVisiontmFLEX High pH, code number K8000, Dako, Denmark) for 60 min at room temperature, immune complexes were visualized by the DacoREALEnVisiontmDetection System (Dako, Denmark). Mayer’s hematoxylin was used for counterstaining of all immune-labeled sections.

### 2.4. Quantitative Image Analysis

For morphological analysis, 30 histological slides per part of colon were examined, both in the control and in the diabetic group. The images for quantitative image analysis were obtained on an Olympus BX50 light microscope equipped with a Leica DFC 295 digital camera (Leica Micro-System, Reuil-Malmaison, France). The photomicrographs were taken at the magnification ×200.

Numerical areal density (N_A_) of IPCs and ICC-IM, i.e., the average number of cells per mm^2^ of the circular and longitudinal muscle layers, was determined with digital image analysis using the ImageJ software (National Institute of Health, Bethesda, MD, USA; http://imagej.nih.gov/ij/ (accessed on 18 December 2022)). N_A_ was determined by using the following formula: N_A_ = (10^6^ × N)/A, (N—number of the cells counted in muscle layer in the visual field, A—area of the muscle layer in mm^2^). We counted the number of these cells in the whole muscular layer in each slide. The cells were counted manually in order to avoid c-kit positive mast cells, which differ from ICC by their shape, granular content and location.

ICC-MP assessment was carried out by estimating the ICC-MP score (percentage encirclement of the ganglion by the processes of ICC-MPs), the semiquantitative method proposed by Den Braber-Ymker [30].

The analysis of the distribution of nerve elements was accomplished by determining the volume density of NF-M-positive fibers in the muscle layer. The volume density (Vv) is a relative variable, which shows how much overall space is occupied by the observed space in volume units. The Vv of nerve fibers and ganglia was determined by using ImageJ and a plugin of the software which inserted a grid system with 336 points (Vt). The number of points overlapping the nerve fibers and ganglia (Vf) within the colon muscle layer was counted. The Vv was determined using the following formula: Vv = Vf/Vt. The obtained results were multiplied by 100 and presented in percentages. In addition, the thickness of the circular and longitudinal muscle layer was determined using ImageJ software.

### 2.5. Statistical Analysis

Statistical analysis was performed using SPSS Statistics (version 20, SPSS, Chicago, IL, USA). The obtained values were compared using the Kruskal–Wallis test with Mann–Whitney U post hoc test.

## 3. Results

Animals in the diabetic group showed significantly higher blood glucose levels (*p* < 0.001), and lower serum insulin levels (*p* < 0.05) compared to the control group (Table 2). In addition, in the STZ–NA group, moderate polydipsia and polyphagia was observed with a significantly higher final body weight (*p* < 0.05).

**Figure 1:**
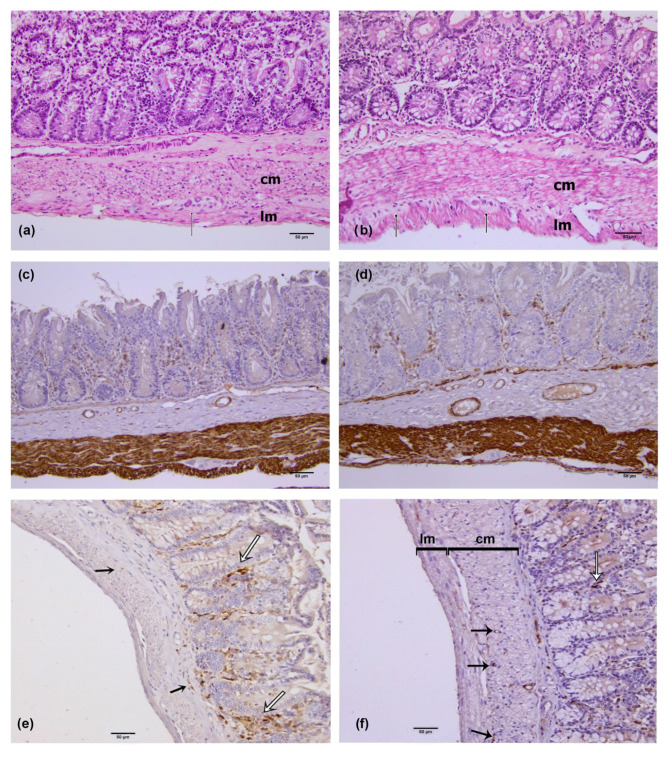
HE (**a**,**b**), desmin (**c**,**d**) and PDGFRα (**e**,**f**) immunohistochemistry. In the muscle layer of both groups (**a**,**b**), ganglia of the myenteric plexus (black arrow) were observed at the border of the circular and longitudinal sublayers. Inside the muscle layer, there were no signs of lymphocytic infiltration, apoptosis or necrosis (**a**,**b**). Desmin-immunoreactivity was present in the wide circular and the thin longitudinal muscle layer of control (**c**) and STZ–NA (**d**) rat colon. PDGFRα immunoreactive cells (**e**,**f**) in the submucosa (white arrow) and within the muscular layer (black arrow), lm (longitudinal muscle layer) cm (circular muscle layer). (**a**) proximal colon of control group; ×200. (**b**) proximal colon of STZ–NA group; ×200. (**c**) proximal colon of control group; ×200 (**d**) proximal colon of STZ–NA group; ×200 (**e**) distal colon of control group; ×200. (**f**) distal colon of the group with diabetes; ×200.

Desmin immunoreactivity was present in the circular and longitudinal muscle layers of the rat colon wall (Figure 1a,b). In the STZ–NA group, there was no difference in muscle wall thickness compared to the control group (Table 3). Further, in the STZ–NA rat colon, there were no signs of necrosis or apoptosis and no evidence of neutrophil or lymphocyte infiltration.

PDGFRα immunoreactivity was present in the mucosa, submucosa, and within the muscle layer of colon segments in both experimental groups. Intramuscular immunoreactive PDGFRα were elongated, spindle-shaped cells, oriented in parallel to the longitudinal axis of smooth muscle cells within the circular colon muscle layer, and they corresponded to IPC (Figure 1c,d—black arrow). These cells were mainly localized on the border between the circular and longitudinal muscle layer and were sporadically distributed in the muscle layer equally in both experimental groups, with a greater presence in the distal colon muscle layer, as shown by the values of the numerical areal density (NA) of IPC (Figure 3). Densely distributed PDGFRα-immunopositive cells were present in the mucosa and submucosa in both experimental groups. Mucosal and submucosal PDGFRα immunoreactive cells had a flat stellated-shaped appearance within the lamina propria, and around and below mucosal colon glands (Figure 1c,d—white arrow).

**Figure 2:**
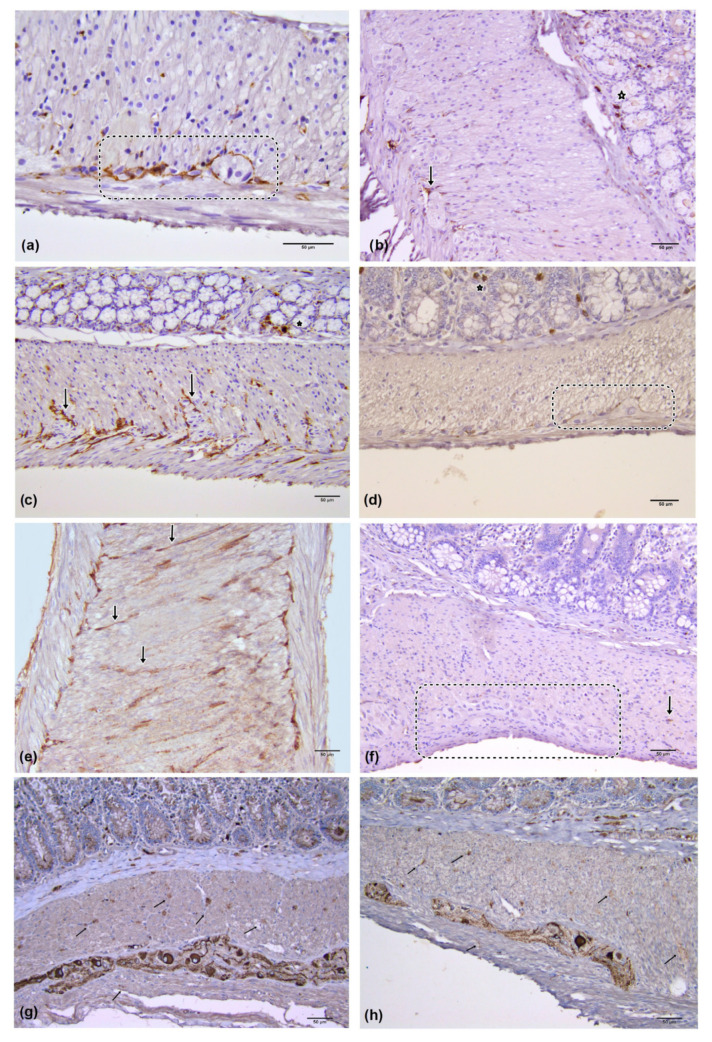
c-kit (**a**–**f**) and NF-M (**g**,**h**) immunohistochemistry. (**a**) longitudinal section of the proximal colon of control group; ICC-MP were densely distributed at the boarder of the circular and longitudinal muscle sublayer and around the MP ganglion (dashed line) ×400; (**b**) longitudinal section of the proximal colon of the group with diabetes mellitus, rare ICC-IM, single ICC-MP cell (arrow) present around the ganglion and c-kit immunoreactive mast cells in the mucosa (asterisk) ×200; (**c**) longitudinal section of the circular muscle layer of control group distal colon, ICC-IM (arrow) were numerous, c-kit immunoreactive mast cells in the mucosa (asterisk) ×200; (**d**) longitudinal section of the distal colon of the group with diabetes mellitus, partial surrounding of MP ganglion by cytoplasmic processes of ICC-MP (dashed line), c-kit immunoreactive mast cells in the mucosa (asterisk) ×200 (**e**) longitudinal section of the caecum of control group; most commonly c-kit IR ICC-IM were single, but also merged into long linear arrays (arrow) ×200; (**f**) longitudinal section of the caecum of the diabetic group, single ICC-IM in the circular muscle layer (arrow), ganglion MP and almost complete absence of ICC-MP with its cytoplasmic processes (dashed line); ×200 (**g**) NF-M IR MP ganglia and nerve fibers (arrow) in control group proximal colon ×200; (**h**) nerve fibers (arrow) and ganglia of the myenteric plexus in distal colon of the diabetic group ×200. Numerical values presented in the figure correspond to the mean values of the N_A_ of IPC and N_A_ of ICC-IM, * statistical significance (*p* ≤ 0.001).

**Figure 3:**
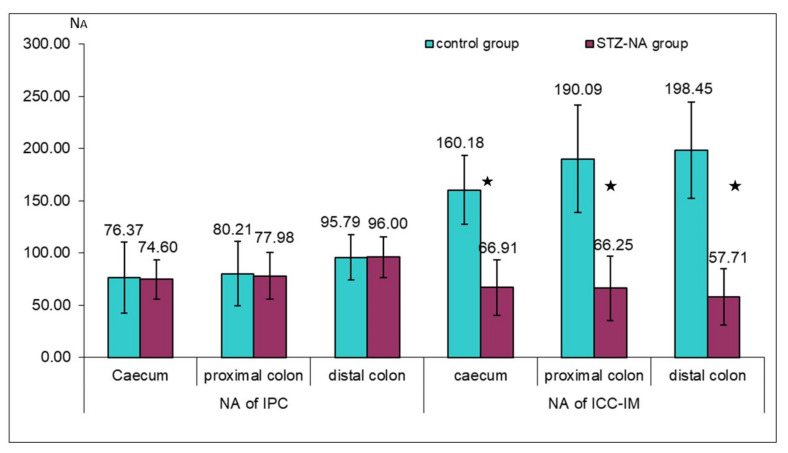
Numerical areal density (N_A_) of IPC and ICC-IM in the circular muscle layer of the rat colon in control and STZ–NA group. Numerical values presented in the figure correspond to the mean values of the NA of IPC and NA of ICC-IM, ★ statistical significance (*p* ≤ 0.001).

Immunoreactive c-kit cells were present within colon muscle layers in both the control and diabetic group. In the control group, immunoreactive c-kit cells identifiable as ICC-IM were seen as spindle-shaped, bipolar cells, situated in parallel to the longitudinal axis of the smooth muscle cells, with two long processes originating from the opposite poles. They were often merged into long linear arrays (Figure 2a,c,e). In the diabetic group, ICC-IM showed the same morphology. However, they were mostly seen as single cells or were completely absent from the visual field (Figure 2b,d,f). The values of the numerical areal density (N_A_) of ICC-IM in examined parts of the colon between the groups showed a statistically significant difference, indicating a significantly reduced number of these cells in the diabetic group (caecum control group N_A_ICC-IM = 160.18 ± 32.83 versus diabetic caecum group N_A_ICC-IM = 66.91 ± 26.80; control proximal colon N_A_ICC-IM = 190.09 ± 51.38 versus diabetic control proximal colon N_A_ICC-IM = 66.25 ± 30.72; control distal colon N_A_ICC-IM = 198.45 ± 46.25 versus diabetic control proximal colon N_A_ICC-IM = 57.71 ± 26.85; *p* ≤ 0.001) (Figure 3).

**Figure 4:**
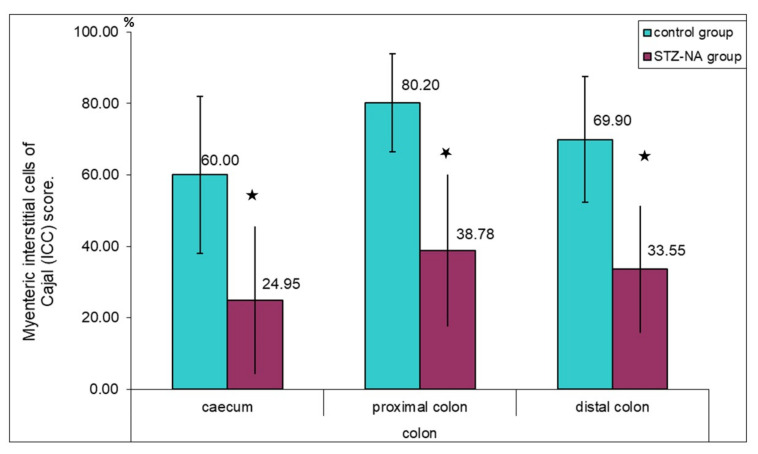
Myenteric interstitial cells of Cajal (ICC-MP) score in the muscle layer of the rat colon in control and STZ–NA group. Numerical values presented in the figure correspond to the mean values of the ICC-MP score, ★ statistical significance (*p* ≤ 0.001).

In both groups, immunoreactive c-kit cells identifiable as ICC-MP presented themselves as multipolar cells whose cytoplasmatic processes were located around the myenteric plexus ganglia (Figure 3a,b). In the control group these cytoplasmatic processes almost completely surrounded the myenteric plexus ganglion, while in the diabetic group it was observed that these cells surround the ganglion only partially. The values of the ICC-MP score in different parts of colon showed a statistically significant difference in the percentage encirclement of the ganglion by the processes of ICC-MP (*p* ≤ 0.001), possibly indicating a decrease in ICC-MP cytoplasmatic processes or their number in the diabetic group, as shown in Figure 4. Individual values of the ICC-MP score were: caecum ICC-MP score = 60.00 ± 21.99 versus diabetic caecum group ICC-MP score = 24.95 ± 20.67; control proximal colon ICC-MP score = 80.20 ± 13.71 versus diabetic control proximal colon ICC-MP score = 38.78 ± 21.31; control distal colon ICC-MP score = 69.90 ± 17.56 versus diabetic control proximal colon ICC-MP score = 33.55 ± 17.74.

In addition to ICC, immunoreactive c-kit mast cells were also found in both experimental groups, and they were clearly different from ICC in their size, shape, lack of processes, and location. Mast cells were located predominantly in the colon mucosa between glands, in the submucosa and within the connective tissue septae of the muscle layers (Figure 2b–d).

**Figure 5:**
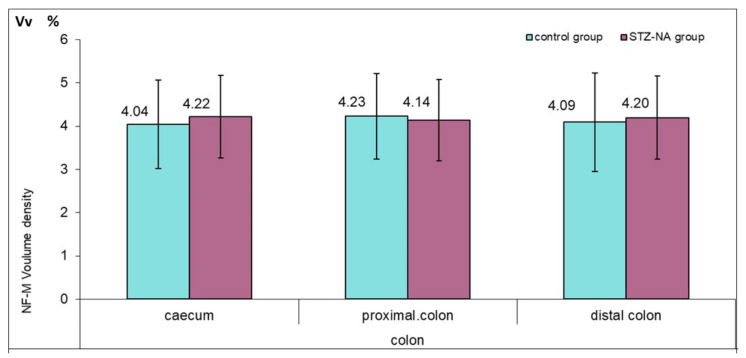
Volume density of NF-M positive fibers in the muscle layer of the rat colon in control and STZ–NA group. Numerical values presented in the figure correspond to the mean values of the NF-M volume density.

On the longitudinal sections of the cecum, proximal and distal colon of the control and STZ–NA groups, NF-M immunohistochemistry showed the presence of ganglion cells of the myenteric (MP) (Figure 2g,h). There was no difference between the groups in the distribution and morphology of MP ganglia. NF-M positive nerve fibers were present in both muscle layers of both experimental groups. There were no statistically significant differences in the volume density of nerve fibers between the groups nor between the different anatomical localizations of the colon within the group (Figure 5).

## 4. Discussion

Hyperglycemia is identified as a risk factor for the development of diabetic neuropathy, including gastroenteropathy [31]. The streptozotocin–nicotinamide animal model is characterized by mild non-fasting hyperglycemia and slightly decreased insulin levels [32], as also shown in our study. Compared to previous animal models which examined ICC loss in diabetes (STZ alone induced diabetes model, NOD mice) [33,34,35,36], advantages of this animal model are that this STZ–NA induced diabetes was more similar to human type 2 diabetes, animals showed moderate hyperglycemia and did not require exogenous insulin to survive.

Intramuscular immunoreactive PDGFRα-positive cells were found to occupy the same intramuscular niches as ICC, but their density in the colon was lower than that of ICC. This finding is similar to that described by Iino et al. within the murine GI tract, where IPC strongly overlapped with ICC [10]. In the present study, there was no significant difference in the density of IPC between the groups, as described by Grover et al. [13]. On the contrary, a high abundance of PDGFRα+ cells in the colon muscle layer in STZ-induced type 1 diabetes was observed in some studies [37,38]. In addition, in both experimental groups there were mucosal and submucosal PDGFRα-immunopositive cells, without any differences in their density and morphology. Kurahashi et al. described a special type of PDGFRα-immunopositive cell in the lamina propria of the human GIT [39]. They suggested that subepithelial PDGFRα-immunopositive cells played a role in sensory and secretomotor signaling, proliferation, differentiation, and apoptosis of epithelial cells, including their pathological inflammatory responses and tumorigenesis. These cells are thought to have modulatory roles in immune and sensory responses and in maintaining mucosal homeostasis. However, the roles of these cells in physiological and pathophysiological processes, such as diabetes, are still unknown. PDGFRα-immunopositive cells have also been found in the subepithelial layer of GIT in adult guinea pig [40] and have been found to play important roles in the morphogenesis of small intestine villi in mice [41].

The c-kit-immunopositive ICC-IM cells were found to be densely distributed throughout the circular and longitudinal muscle layer in the cecum and proximal and distal colon in the control group. Such a distribution of ICC-IM, with the highest value of the numerical areal density (NA) in the descending colon, was similar to that seen in the human and rat colon [42,43]. There was a significant loss of ICC-IM and ICC-MP in the caecum and proximal and distal colon of the experimental group with diabetes mellitus type 2. In rats with diabetes, there was a decrease in the degree of networking of all ICC subtypes. The absence of linear ICC-IM cell connections, and reduction in the number of ICC-IM in the circular muscle layer, is believed to contribute to the symptomatology of diabetic gastroenteropathy due to impaired inhibitory neurotransmission [44,45]. Furthermore, ICC-IM are important in transmitting mechanical activity throughout the musculature in the proximal colon to the autonomic nervous system [6,45]. Wang et al. described a nerve fiber loss due to ICC-IM loss in the experimental group with diabetes [33]. The loss of nerve fibers was caused by the loss of ICC-IM and was partially due to depletion of synapse-like connections with ICC-IM. These findings emphasize the importance of ICC and also show how ICC alterations occurring in diabetes can be one of the major factors in the development of gastroenteropathy. However, in this study, there was no nerve loss or morphological changes in smooth muscle cells. We therefore can assume that during the development of peristaltic complications in diabetes, the loss of ICC occurs first, followed by a reduction in nerve fibers. The interconnectedness and interdependence of nerves, ICC, and smooth muscle cells is explained by SCF, which is necessary for the survival of ICC and is secreted locally by nerves and muscle cells. The decline in the number of ICC and impairment in the ultrastructure of ICC are attributed to a deficiency in the endogenous SCF, but are not related to hyperglycemia [35]. It is possible that the loss of ICC, without observed loss of nerve fibers, is due to impaired intracellular SCF signaling and the sensitivity of ICC to reduced insulin levels in this diabetes model.

Reduction in the ICC-MP score and the networking of ICC-MP around ganglia observed in this study in the diabetic group can affect the frequency and propagation velocity of peristalsis proposed by Huizinga et al. [28]. These cells are the dominant pacemaker cells generating rhythmic depolarization, and an ICC-MP score of less than 30% in the small intestine and 10% in the large intestine is considered pathological [30]. The values of the ICC-MP score for the colon are lower due to the additional network of submucosal ICC in the human colon. In this study, ICC scores were 24.95 ± 20.67 in the caecum; 38.78 ± 21.31 in the proximal colon and 33.55 ± 17.74 in the distal colon, which was insufficient for peristalsis disorder according to the International Working Group on gastrointestinal neuromuscular diseases [46]. It should be noted that, there is no additional submucosal ICC network in the rat colon.

Hyperglycemia-related oxidative stress is thought to be the initial step in the development of diabetic complications. Disorder and reduction in ICC in STZ–NA-induced diabetes mellitus can be caused by hyperglycemia and increased oxidative stress, insulin and insulin-like growth factor deficiency, autoimmune response, or a combination of these factors [21,47]. Increased oxidative stress associated with hyperglycemia has been reported in diabetic NOD mice with gastroparesis [47]. According to Choi et al., along with oxidative stress in diabetes mellitus, there is a reduction in neural NO synthase and hem oxygenase-1 (HO-1) expression, which are potentially cytoprotective and a survival factor for ICC [48]. This finding emphasizes the interdependence between ICC and nerve fibers, as discussed previously. However, Horvat et al. have shown that hyperglycemia is not enough to cause changes in ICC, but that instead the decrease in insulin and insulin-like growth factor (IGF-1) signaling in diabetes played a major role in reduced ICC survival [49]. Ordog et al. reported that insulin and IGF-1 completely prevented the loss of ICC in the murine gastric tunica muscularis and the loss of ICC and nerve structures [50]. In addition, in our recent paper [51], treatment with antioxidants that normalized hyperglycemia in STZ–NA-induced diabetes type 2 had no effect on the preservation of ICC, indicating that insulin signaling, which is deficient in diabetes, was the main cytoprotective factor for the survival of ICC.

ICC and IPC represent two classes of interstitial cell with a unique ultrastructure, molecular phenotype, and function. Smooth muscle cells are electrically connected to ICC and IPC by gap junctions and form an integrated unit—smooth muscle cell, ICC and PDGFRα-immunopositive cell (SIP) syncytium [52]. SIP cells express different receptors and ion canals, and changes in the conductivity of any type of SIP cell type affect excitability and syncytium responses. Any alteration of the SIP syncytium cells may lead to the colonic motor dysfunction seen in diabetic gastroenteropathy. Functional defects associated with diabetic gastroenteropathy, such as a weak muscular response to enteric neuron activation, decreased or arrhythmic slow wave activity of the intestine, and colonic motor dysfunction requiring surgery, have been associated with changes in the number of ICC and IPC [13,26,28,37,53,54]. The imbalance between loss of ICC and IPC proliferation caused by hyperglycemia in the colonic SIP syncytium leads to diabetic slow-transit constipation [55]. In this paper, a loss of ICC-IM with no ICC-IM merged into long linear arrays was observed, along with an ICC-MP score reduction in rats with diabetes mellitus type 2 without changes in IPC distribution. Bearing in mind the mechanism of SIP syncytium functioning, these cellular variations can lead to diabetic disturbances of peristalsis in the colon.

Activation of autoimmune response is just one more effect of diabetes on interstitial cells, as the immune system cells may be involved in their loss and degenerative changes. Our study showed no signs of apoptosis or lymphocytic and neutrophilic infiltration, but numerous mast cells were observed. Mast cells were present mainly in the mucosa between the glands and in the connective tissue of the submucosa in both experimental groups equally. In human studies of Crohn’s disease and achalasia, a close contact between “injured” ICC and mast cells was found [56,57]. However, although the results of this study showed a higher number of mast cells in both experimental groups equally, there was a loss of ICC density in rats with diabetes, which suggested that a disorder of growth factors secreted by mast cells could have affected the preservation of the ICC.

## 5. Conclusions

In conclusion, a statistically significant decrease in the number of the intramuscular ICC and myenteric ICC was observed in all examined parts of colon in rats with type 2 diabetes. The nerve fiber distribution and volume density did not show the differences between the control and diabetic group. IPC were rarely present within the colon muscle layer, with densely distributed PDGFRα+ cells in the colon mucosa and submucosa in both experimental groups. A loss of ICC-IM and ICC-MP might play a role in the pathogenesis of diabetic gastroenteropathy, and due to their role in intestinal motility, ICC can be a significant link in the development of more effective therapies for diabetic gastroenteropathy.

## Figures and Tables

**Table 1:** Antibodies.

Primary Antibody	Supplier	Dilution	Incubation
PDGFRα (Rabbit polyclonal anti-PDGFR alpha antibody)	Abcam (Cambridge, UK) Ab65258	1:100	1.5 h at room temperature
C-kit (Rabbit monoclonal anti-c-Kit antibody)	Abcam (Cambridge, UK) Ab32363	1:100	24 h overnight at 4 °C
NF m (Rabbit polyclonal anti Neurofilament Medium antibody)	Abcam (Cambridge, UK) Ab9034	1:1000	24 h overnight at 4 °C
Desmin (Monoclonal Mouse Anti-Human Clone D33)	Daco (Denmark) MO760	1:100	1.5 h at room temperature

**Table 2:** Average initial and final body weight, glucose and insulin levels in control (C) and diabetic (STZ–NA) rats.

Parameters	Groups	
	Control	STZ–NA
Initial body weight (g)	351.7 ± 7.5	352.5 ± 14.9
Final body weight (g)	395.0 ± 12.3	413.7 ± 19.2 **
Blood glucose levels (mmol/L)	6.36 ± 0.01	12.02 ± 1.65 *
Serum insulin levels (pmol/L)	217.1 ± 21.2	191.2 ± 16.3 **

Values are mean ± S.D. of the mean; * statistical significance (*p* ≤ 0.001), ** statistical significance (*p* ≤ 0.05).

**Table 3:** Thickness of circular and longitudinal muscle layer in control and diabetic (STZ–NA) rats.

Group	Caecum		Proximal Colon		Distal Colon	
	Circular Muscle Layer	Longitudinal Muscle Layer	Circular Muscle Layer	Longitudinal Muscle Layer	Circular Muscle Layer	Longitudinal Muscle Layer
CONTROL	174.015 ± 25.57	25.786 ± 5.34	160.152 ± 28.41	23.85 ± 3.605	156.152 ± 26.23	26.11 ± 4.77
STZ–NA	178.441 ± 35.57	25.719 ± 5.42	155.786 ± 27.31	23.208 ± 5.12	155.786 ± 27.31	25.91 ± 4.76

Values are mean ± S.D. of the mean.

## Data Availability

The data presented in this study are available on request from the corresponding author.
